# Artificial Neural Networks for Impact Strength Prediction of Composite Barriers

**DOI:** 10.3390/ma18133001

**Published:** 2025-06-24

**Authors:** Yuyi Zhang, Andrey Logachev, Aleksandr Smirnov, Nikita Kazarinov

**Affiliations:** 1 Faculty of Mathematics and Mechanics, Saint Petersburg State University, Saint Petersburg 199034, Russia; lesliezhang0825@gmail.com (Y.Z.); a.n.logachev@mail.ru (A.L.); alex.smirnov318@gmail.com (A.S.); 2 Research Center of Dynamics, Institute of Problems of Mechanical Engineering, Saint Petersburg 199178, Russia

**Keywords:** impact, composite, FEM, neural network, incubation time

## Abstract

This study considers the impact and penetration of composite targets by steel projectiles. Firstly, experiments on the impact of homogeneous polymethyl methacrylate (PMMA) targets were simulated using the finite element method (FEM) and the incubation time fracture criterion (ITFC). Next, targets were assumed to be composed of cells with weakened mechanical properties, forming a composite barrier. The composite impact problems were then used to demonstrate an approach, which can be applied to overcome the typical difficulties for impact simulations—high demands on computing resources, long computation times, and potential numerical instabilities arising from high stresses in the contact zone and high strain rates. The approach is based on the use of artificial neural networks (ANNs) trained on arrays of numerical results obtained via finite element method.

## 1. Introduction

In recent years, neural networks, particularly artificial neural networks (ANNs) and convolutional neural networks (CNNs), have gained significant traction in addressing a wide range of problems in engineering and mechanics [1,2,3,4,5]. Their applications have been especially notable in fluid dynamics [6,7,8,9,10] and solid mechanics [11,12,13,14]. When integrated with numerical methods, neural networks have proven effective in solving complex engineering challenges. They have been successfully applied to optimize shape design [15,16], estimate material properties [17,18], and tackle intricate mechanical systems [19,20] that are otherwise computationally intensive. These advancements have not only expanded the role of machine learning in engineering but have also enhanced the efficiency of design and optimization processes. This is particularly valuable in scenarios involving high complexity or large-scale simulations. CNNs have demonstrated significant promise in solid mechanics, particularly in the design and optimization of composites [21,22], metamaterials [23,24], nanomaterials [25], and other complex structures [26]. One key advantage of CNNs lies in their ability to effectively capture and extract features from multidimensional data [27]. This capability is essential for training accurate models and plays a critical role in optimizing network performance. Compared to traditional ANNs, CNNs offer superior representational power, making them especially well-suited for handling the intricate data structures common in material science [28]. Additionally, the inherent architecture of CNNs enables them to address challenges such as cross-scale analysis [29] with greater ease. This structural advantage leads to improved efficiency and accuracy in material design, streamlining the development process for advanced mechanical systems.

Despite their advantages, CNNs also present several limitations. Their performance is heavily dependent on the quality and distribution of input data. When datasets are limited or unevenly distributed, CNNs are prone to overfitting, performing well on training data but poorly on new or unseen samples [30,31]. This issue becomes especially pronounced in complex mechanical systems, where extensive experimental or simulation data may be unavailable. To mitigate these challenges, researchers have explored various enhancements [32,33]. Deeper architectures and alternative network designs, such as residual networks (ResNets) [34,35], have been introduced to improve feature extraction across multiple scales and to enhance the generalization performance.

The increasing computational power and the rapid expansion of data volumes have made the integration of machine learning with traditional numerical methods, such as the finite element method (FEM) and finite difference method (FDM), a vital approach in engineering mechanics [36]. Traditional numerical methods are often resource-intensive, requiring substantial computational time and power, particularly when solving complex engineering problems involving large-scale structures or dynamic loading conditions [37]. While these methods can yield precise solutions, their high computational cost and resource demands remain significant challenges [38]. As a result, recent research has focused on integrating machine learning techniques with classical methods to enhance the computational efficiency without compromising the accuracy [39]. A common strategy is to leverage machine learning models to replace certain parts of the numerical calculation process [40]. In finite element analysis, for instance, iterative meshing, solving mechanical equations, and repeated calculations are typically required. By utilizing machine learning models, such as neural networks, researchers can train predictive models that directly estimate a structure’s response from design parameters or initial conditions, thereby bypassing many of these time-consuming iterative steps [41]. This approach can drastically reduce computation times, particularly in areas like structural optimization, shape optimization, and material design [37]. In these cases, machine learning models can provide quick approximations, which can then be refined by traditional numerical methods, thus striking a balance between efficiency and accuracy [36].

The central focus of this study is the prediction of the impact strength of perforated polymethyl methacrylate (PMMA) targets. Accurately predicting impact strength is crucial for design and material selection in various engineering fields, including aerospace, military protection, and materials science. Understanding the damage behavior of materials under impact loading has become a key area of research. Traditional methods, such as FEM-based simulations, can provide precise mechanical responses and damage modes. However, these methods are often limited in real-time applications and efficient optimization due to their high computational cost and time requirements. As a result, developing a machine learning model capable of quickly and accurately predicting impact strength is of significant practical value. The occurrence of perforation is influenced by several factors, including the projectile velocity, target thickness, and material properties. Traditional numerical simulations require extensive calculations for various conditions, which can be time-consuming. To address this challenge, this study proposes a deep learning approach, specifically, a CNN, trained on a dataset generated by FEM simulations. This method allows for the rapid prediction of the impact strength, enabling faster analysis of perforated PMMA targets under different conditions.

During our experiments, we observed that the residual velocity of the projectile often displayed negative values when the projectile velocity was below 65 m/s. This resulted in a bimodal distribution of the data. Such a distribution poses a challenge for neural network training, as standard machine learning models typically assume a unimodal distribution. This can lead to an insufficient understanding of the data, potentially reducing the prediction accuracy. To address this issue, we introduced a mixed density network (MDN) in the output layer and developed a new ResNetMDN architecture by integrating the residual connectivity (ResNet) mechanism. The MDN is well-suited for handling complex multimodal distributions and is particularly effective for addressing bimodal patterns. Meanwhile, the ResNet mechanism helps mitigate issues such as vanishing or exploding gradients in deeper networks by incorporating jump connections, thus enhancing both the training efficiency and stability. With this novel network architecture, we successfully built an efficient prediction model capable of quickly estimating the perforation strength of PMMA targets under a range of impact conditions. Notably, the model performs exceptionally well under high-speed impacts, such as when the projectile velocities exceed 144 m/s or 199 m/s. It not only achieves a prediction accuracy comparable to traditional FEM simulations but also significantly outperforms these methods in computational speed, making it a valuable tool for real-time monitoring and rapid optimization in practical engineering applications.

Simulation failures are a common challenge in FEM, particularly when complex mechanical behaviors are involved. FEM works by discretizing a structure into small cells through meshing and computing the mechanical response of each cell. However, when large deformations, contact issues, or material failures occur, traditional FEM simulations can fail due to mesh distortion, numerical instability, or convergence problems during iterations. In this study, we leveraged a pre-trained neural network to predict instances where FEM simulations are likely to fail, specifically focusing on the predicted values of projectile residual velocities for these cases. For these predicted failure instances, we applied a fine-tuning strategy to reprocess the data. The corrected FEM simulation results were then used as labeled values to validate and evaluate the neural network’s prediction performance. This approach allowed us to effectively assess the neural network’s accuracy in handling complex real-world scenarios. The main contributions of this study are as follows:

- Development of the ResNetMDN Architecture: This study introduces a novel neural network architecture that combines the residual connectivity mechanism (ResNet) with a mixed density network (MDN) output layer. This design effectively handles data with bimodal distributions, addressing the limitations of conventional neural networks in predicting the impact intensity.
- Optimization of Impact Strength Prediction: By utilizing a training dataset generated through finite element method (FEM) simulations, a deep learning model is developed to rapidly and accurately predict the impact strength of perforated PMMA targets under varying projectile velocities. This approach significantly enhances both the computational efficiency and prediction accuracy.
- Addressing FEM Simulation Failures: The trained neural network is employed to predict instances where FEM simulations may fail, enabling more reliable predictions and improving the overall robustness of the model.

## 2. Materials and Problem Statement

In this section, the problem is described. We studied the penetration of composite barriers by steel projectiles using FEM and artificial neural networks. We assumed that the basic target was made of PMMA (polymethilmetacrilate), and the residual velocity of the projectile was studied as the indicator of the target performance—the lower the residual, the stronger the barrier. Firstly, we describe the solution to a basic problem: we used the FEM together with the incubation time fracture criterion (ITFC) to simulate penetration of the PMMA target by steel projectiles at various velocities and calibrated our results against the real impact experiments. Then, we assumed that the target was assembled from parts with varying mechanical properties and we solved a corresponding set of problems with varying composition of targets. This set of solutions was then used to train artificial neural networks, which can predict the solution for another representative of the aforementioned family of problems. In the following subsections, more details are provided.

### 2.1. Problem Statement

This study addresses a class of problems involving *M* composite configurations, where each problem is defined by a parameter vector $P^{j}=\left(P_{1}^{j},P_{2}^{j},\ldots ,P_{N}^{j}\right)$, with j∈[1,M]. Traditionally, each configuration $P^{j}$ is solved using the finite element method (FEM) or other numerical schemes to obtain the corresponding result $R^{j}$. However, in certain cases, the numerical scheme may fail due to specific parameter combinations, resulting in no output, denoted as $R^{j}=⌀$. These failure cases are explicitly included in the dataset:

(1) $$\{\;P^{j}\to R^{j}|j\in \left[1,M\right]\}.$$

The objective of this research is to develop an algorithm capable of predicting the result $R^{M+1}$ for a new unsolved composite configuration $P^{M+1}$, without performing a full numerical simulation. Importantly, the algorithm should also be capable of handling cases where traditional numerical methods fail to produce results ($R^{j}=⌀$). To achieve this, the study proposes the use of artificial neural networks (ANNs) as predictive models. The approach involves training the ANN on the subset of configurations for which valid numerical results are available. Once trained, the network is then used to infer approximate results for previously unsolvable configurations. Each parameter vector $P^{j}$ may describe aspects such as the sample geometry, material properties, loading conditions, or manufacturing processes. The dataset size *M* is assumed to be sufficient to ensure the convergence and effectiveness of the neural network training.

From a mechanical perspective, the problem can be formulated as follows: a circular PMMA plate with a diameter of 100 mm and a thickness of 10 mm is impacted by a cylindrical steel projectile (the material properties are listed in Table 1). The projectile has a diameter of 6.9 mm and a height of 30 mm. To simplify the modeling process, a two-dimensional axisymmetric model is employed, in which both the target and the projectile are represented as rectangular domains. Fixed boundary conditions are applied to the edges of the target plate.

**Figure 1:**
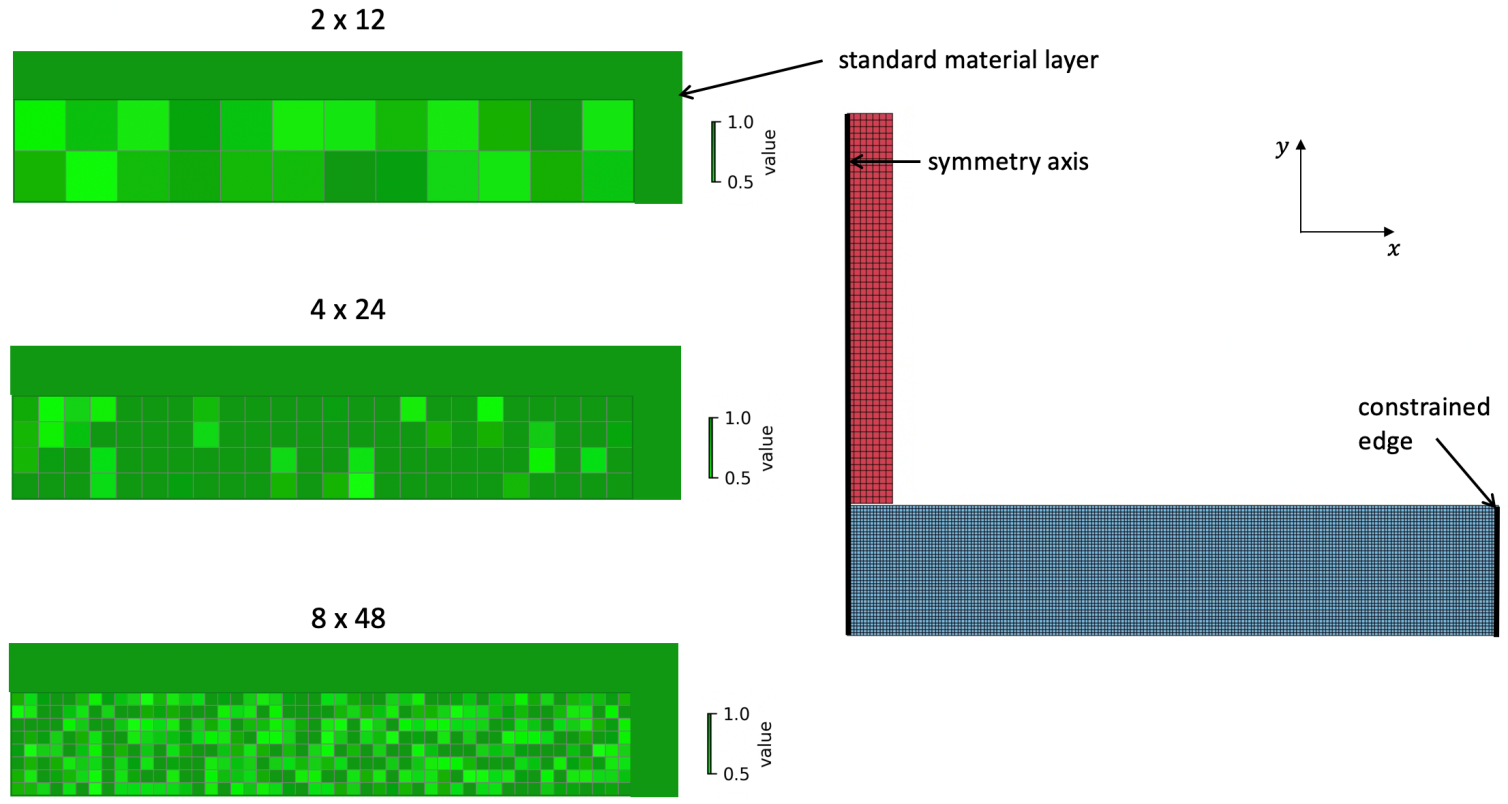
Representatives of the three composite configuration sets: 2 cells × 12 cells, 4 × 24, 8 × 48. All the composite configurations have a layer of standard material.

During the impact event, the projectile decelerates due to its interaction with the target, and its residual velocity is determined through numerical simulation. Since the deceleration behavior is strongly influenced by the structural configuration of the composite material, an artificial neural network (ANN) can be employed to predict the residual velocity of the projectile under various composite configurations. This approach provides a useful tool for material design and protective performance evaluation. In this study, three resolutions of the composite configurations were examined: 2 cells × 12 cells (2 × 12), 4 × 24, and 8 × 48. Additionally, the plate was assumed to have a layer of standard PMMA for contact stabilization. See Figure 1 for examples of the composite configurations. The material properties of selected cells (Young’s modulus, ultimate stress, and incubation time) are decreased for each individual cell by multiplier from a range.

### 2.2. Materials

**Figure 2:**
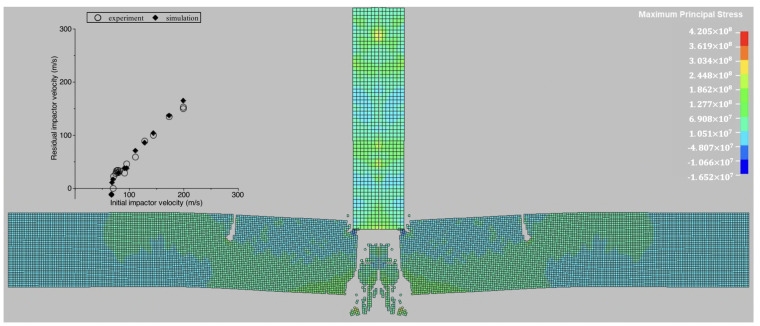
The FEM model calibration—homogenous target composite configuration; experimental results from [42].

As mentioned above, the PMMA was used as the material for the basic problem: the impact of a homogeneous PMMA target by a steel cylindrical plunger. The FEM model performance was calibrated against some experimental data: the experimental and numerical results for the homogeneous target are shown in Figure 2. The standard and modified PMMA properties are listed in Table 1.

## 3. Methods and Techniques

Here, the numerical approaches we used to generate datasets and the tested ANN architectures applied for the residual velocity prediction are described.

### 3.1. The FEM Model

The impact problem under investigation was modeled and simulated using the LS-DYNA R14.1.0 solver, a commercial finite element analysis software developed by Livermore Software Technology Corporation (Livermore, CA, USA). LS-DYNA employs an explicit time integration scheme and, in this study, was utilized with a user-defined material model incorporating the incubation time fracture criterion (ITFC) to capture the dynamic fracture behavior accurately. The finite element mesh consisted of square elements with four integration points. For all composite configurations, the mesh was generated using uniformly sized square elements, with the element size determined by the requirements of the fracture model. The finite element model was calibrated against the previously reported experimental data [42]. Figure 2 presents a comparison between the numerical and experimental results, along with the stress distribution at 36 μs for an impactor velocity of 91 m/s.

The dynamic fracture behavior of brittle solids represents a complex phenomenon, necessitating the application of robust and accurate fracture models to ensure reliable simulation outcomes. These models must be capable of capturing rate-dependent fracture characteristics, such as the influence of the loading rate on the material strength (e.g., see [43,44]) and the phenomenon of fracture delay [45,46,47]. Among the most prevalent modeling approaches are those based on stress intensity factors for crack propagation [48], as well as constitutive models that explicitly incorporate strain-rate effects, such as the widely utilized JH-2 model [49]. In the present study, the fracture behavior was analyzed using the incubation time model [50,51], which has demonstrated effectiveness across various dynamic fracture scenarios, including crack propagation [52,53] and solid particle erosion [54], while maintaining relative simplicity. The incubation time approach adopted herein is defined by the following criterion:

(2) $$\frac{1}{\tau }\int_{t-\tau }^{t}\frac{1}{d}\int_{x}^{x+d}\sigma \left(x^{\prime },t^{\prime }\right)dx^{\prime }dt^{\prime }\geq \sigma_{c}.$$

In Equation (2), $\sigma_{c}$ denotes the ultimate tensile strength of the material, and *d* represents a characteristic length scale associated with the minimum size of the fracture process zone. This parameter defines the spatial resolution at which damage is considered significant; zones smaller than *d* are not classified as fractured. The value of *d* is computed via the relation $d=\frac{2K_{IC}^{2}}{\pi \sigma_{c}^{2}}$, where $K_{IC}$ is the critical stress intensity factor. This formulation inherently introduces a non-local perspective to fracture modeling, acknowledging that the process cannot be accurately characterized as occurring at a single point in space. Nevertheless, the fracture process typically initiates at localized regions and evolves through precursor mechanisms, such as the coalescence of microdefects, microcracks, and voids. These mechanisms are effectively captured through the stress history and a characteristic temporal parameter—the incubation time τ. The criterion presented in Criterion (2), formulated as an inequality, is commonly employed for the prediction of brittle fracture. Notably, the incubation time model has also demonstrated considerable success in the context of dynamic plasticity modeling [55].

**Figure 3:**
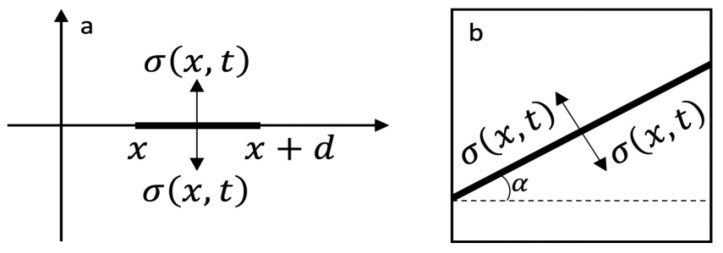
Spatial integration in the incubation time fracture criterion (ITFC). (**a**) interval of spatial integration (incubation time fracture model); (**b**) element with a fracture area.

The proposed model was implemented within the LS-DYNA simulation framework via user-defined material subroutines (UMAT41). Stress histories were recorded in dedicated arrays, and the time integration involved in the fracture criterion (2) was performed using the trapezoidal rule. To ensure a structured and consistent mesh, all simulations utilized square elements of uniform size. The stress values computed at the four integration points within each element were averaged to evaluate the spatial integral in the criterion (2). If the criterion predicted fracture initiation in any of the eight predefined subregions within an element, the element was considered to have failed and was subsequently removed from the mesh. The following area angles were considered (see Figure 3b): $\alpha =0,\frac{\pi }{2},\pm \frac{\pi }{3},\pm \frac{\pi }{4},\pm \frac{\pi }{6}$.

### 3.2. CNN Models

#### 3.2.1. Architecture of the CNN Model

**Figure 4:**
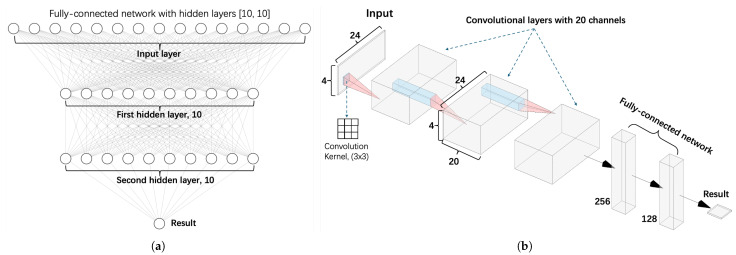
Schematic representation of the neural network architectures. (**a**) illustrates a fully connected network comprising an input layer followed by two hidden layers, each containing 10 neurons, culminating in the output layer. (**b**) depicts a convolutional neural network featuring two convolutional layers, each with 20 channels and a 3 × 3 kernel, followed by fully connected layers that process the extracted features to produce the final output.

This section outlines the convolutional neural network (CNN) architecture employed in our research, as illustrated in Figure 4b. The CNN is a deep learning model designed to process grid-like data by progressively extracting features from simple patterns to more complex structures.

The network begins with a feature extraction module that alternates between convolutional and pooling layers. In the convolutional layers, a 3×3 sliding window scans the input, performing a weighted sum followed by a non-linear activation using the rectified linear unit (ReLU), defined as relu(x)=max(0,x). To ensure stable training, each convolutional layer is followed by a batch normalization layer, which standardizes the output much like dimensionless normalization in mechanical analysis. A maximum pooling layer then downsamples the spatial dimensions by retaining the highest values in each region, analogous to identifying peak stress concentrations.

Following feature extraction, the network shifts to a fully connected prediction module (Figure 4a), which acts similarly to a traditional regression model. Here, the spatial features are flattened into one-dimensional vectors and processed through two fully connected layers. To reduce the risk of overfitting, dropout is applied to randomly disable some connections, akin to introducing simplifying assumptions to filter out experimental noise. The final output layer uses linear activation to directly predict the bullet residual velocity. The training process employs the adaptive moment estimation (Adam) optimizer, which dynamically adjusts the learning rate.

#### 3.2.2. Improved CNN Model: ResNet

**Figure 5:**
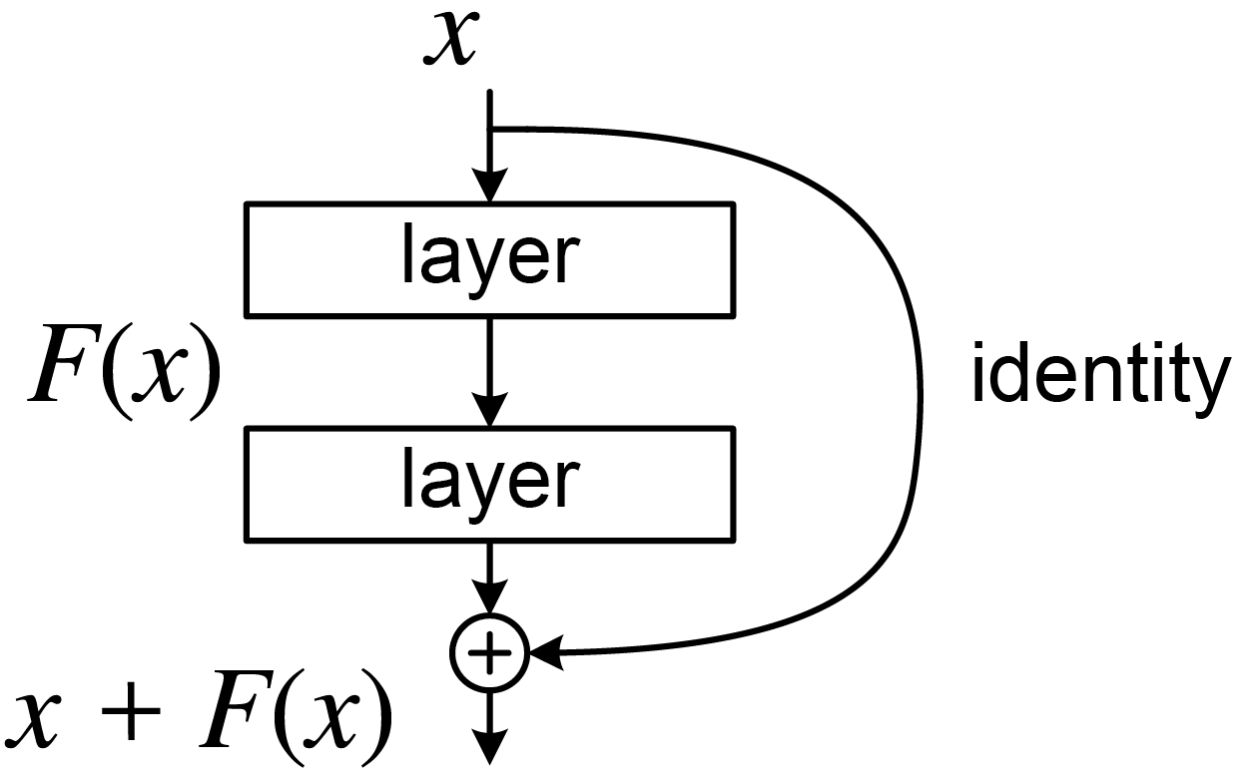
Schematic diagram of the residual block.

The residual network (ResNet) was introduced to address the performance degradation observed in deep neural networks as their depth increases. Traditional networks often suffer from vanishing or exploding gradients when exceeding 20 layers, leading to training instability. ResNet mitigates this issue through residual learning, where each residual block (Figure 5) employs the mapping H(x)=F(x)+x. Here, *x* represents the input features, and F(x) is a residual function consisting of two 3×3 convolutional layers. By incorporating an identity shortcut connection, gradients can propagate directly backward, significantly easing the optimization of deep networks. When the input and output dimensions differ, a 1×1 convolutional layer performs linear projection to align them.

ResNet utilizes a pre-activation structure, arranging layers in the order Conv-BN-ReLU (convolution, batch normalization, ReLU activation). This sequence promotes a more stable gradient flow compared to conventional designs. During spatial downsampling, a convolutional layer with a stride of 2 halves the feature map resolution while expanding the channel depth. To further enhance the feature extraction, dilated convolutions are integrated in deeper layers, enlarging the receptive field without additional parameters by adjusting the dilation rate. A standard ResNet composite configuration stacks 4–6 residual blocks, each containing two convolutional layers, with total depths typically between 20–30 layers to balance the performance and computational efficiency.

In this study, ResNet is particularly advantageous for three key reasons:

1. The residual connections retain the original input features, preventing excessive loss of geometric mesh information in deep layers;
2. The deep structure extracts multi-scale features, from the local mesh morphology to global distribution patterns;
3. Skip connections facilitate gradient flow, enabling stable and effective training even at greater depths.

### 3.3. ResNetMDN

Mixture density networks (MDNs) combine neural networks with probability modeling to capture uncertainty and multiple outcomes in complex data. Instead of producing a single deterministic output, this network predicts the parameters of the probability distributions—often Gaussian mixtures—that represent the range of possible outcomes. The probability density function is given by

$$p\left(y|x\right)=\sum_{k=1}^{K}\alpha_{k}\left(x\right)\;\cdot \;N\left(y;\mu_{k}\left(x\right),\sigma_{k}^{2}\left(x\right)\right),$$

where the neural network outputs a mixture of weights $\alpha_{k}$, mean $\mu_{k}$, and variance $\sigma_{k}^{2}$, and *K* is the number of mixture components.

This approach is especially useful in mechanics, where multiple solutions may arise from the same input conditions. For example, materials subjected to the same load might fail in different ways, or their crack propagation paths could vary due to underlying bifurcation phenomena. By modeling multiple peaks explicitly, MDNs help avoid the bias that can occur when traditional regression methods oversimplify the problem by assuming a single outcome.

The ResNetMDN model integrates residual networks and mixed density networks to address complex regression tasks with a multimodal approach. It begins by using a residual network with cross-layer constant mapping as its feature extractor, capturing high-dimensional representations through layer-by-layer nonlinear transformations. This design mitigates the gradient decay problem and ensures stable feature modeling. In the decoding phase, the model reduces spatial features to low-dimensional vectors using global pooling. These vectors are then processed by a density network that generates weight coefficients, mean vectors, and covariance matrices to form a mixture of Gaussian distributions. This process allows the model to represent multimodal conditional probability distributions effectively.

The differences between ResNetMDN and traditional methods are demonstrated in Table 2. Overall, ResNetMDN offers three key advantages over traditional regression models: robust feature extraction via a deep residual structure, the ability to model multimodal outputs with a hybrid density layer, and enhanced uncertainty quantification through integrated joint optimization.

### 3.4. Evaluation of the Efficiency

In assessing the performance of regression models, the primary evaluation metrics utilized are the coefficient of determination ($R^{2}$) and the mean squared error (MSE). In this study, we focused on two distinct types of data to characterize the perforated targets. The first type employed binary data to indicate the presence of perforations—where 1 denotes a perforated target, and 0 indicates an unperforated one. The second type utilized continuous data, providing a more nuanced representation of the perforated targets.

Given this dual approach, $R^{2}$ serves as the key metric for evaluating the model’s efficiency in predicting the residual velocities of the projectiles. This preference arises from the superior normalization capabilities of $R^{2}$ compared to the MSE. Specifically, $R^{2}$ enables a comparison of the model’s performance against a baseline, such as the mean value, thereby offering a normalized criterion for evaluation. The formula for $R^{2}$ is expressed as follows:

(3) $$R^{2}=1-\frac{\sum_{i=1}^{n}{\left(p_{i}-y_{i}\right)}^{2}}{\sum_{i=1}^{n}{\left(\bar{y}-y_{i}\right)}^{2}}.$$

Additionally, it quantifies the proportion of total variance in the data that is accounted for by the model, with values ranging from −∞ to 1. A higher $R^{2}$ value signifies a better fit; for instance, an $R^{2}$ of 0.8 indicates that 80% of the variance in the data can be explained by the model. Conversely, a negative $R^{2}$ suggests that the model performs worse than simply using the mean of the observations as a predictor, establishing a clear benchmark for assessing model efficiency.

## 4. Results

### 4.1. Data Visualization

**Figure 6:**
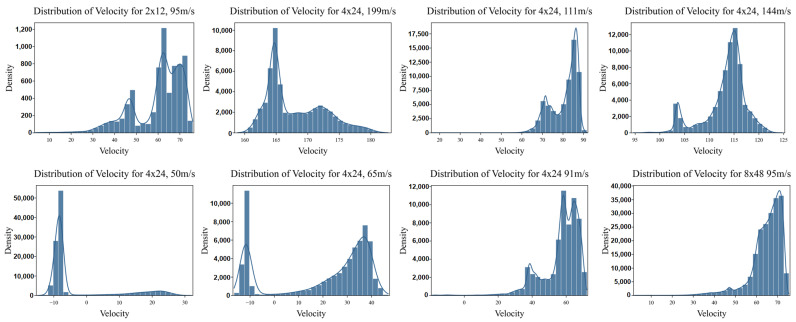
Residual velocity distributions across impact conditions.

The data depict the distribution of the residual velocities *v* of the projectiles after impacting a plate (Figure 6), where the horizontal axis represents the velocity, and the vertical axis denotes the corresponding density ρ(v). For higher initial velocities (e.g., 199 m/s), the distribution is right-skewed, with ρ(v) concentrated in the positive domain, indicating successful penetration. In contrast, at lower initial velocities (e.g., 50 m/s and 65 m/s), a significant portion of the distribution extends into the negative velocity range, reflecting elastic rebound events, where the projectile reverses direction due to insufficient kinetic energy for penetration. This results in a pronounced left-tailed density profile, with ρ(v) exhibiting non-negligible mass for v<0.

### 4.2. Effective Performance of Networks

In this study, we investigated the relationship between the network structure and the prediction accuracy by evaluating the performance of different deep neural network architectures for predicting the projectile residual velocity (Table 3).

The results reveal a complex velocity dependence, with the five-layer asymmetric architecture (20, 40, 80, 40, 20) and the ResNetMDN model offering complementary advantages across various initial velocity conditions.

The ResNetMDN model, featuring a residual connectivity mechanism, demonstrates superior prediction stability in the medium velocity range (95–144 m/s), particularly for a mesh division of 2×12. At an initial velocity of 95 m/s, it achieves an R^2^ of 0.936, largely due to its jump connection that mitigates the gradient vanishing problem, enabling it to better model the nonlinear characteristics of moderate-speed trajectories.

In contrast, the five-layer asymmetric structure (20, 40, 80, 40, 20) excels at both high velocities (199 m/s) and low velocities (50 m/s). At 199 m/s, it produces the highest R^2^ value of 0.935, surpassing the ResNetMDN by 3.2 percentage points. Although at 50 m/s, its R^2^ performance of 0.689 slightly lags behind ResNetMDN’s 0.704, it still outperforms other symmetric architectures. This advantage can be attributed to the structure’s broad receiving domain of 80 nodes in the middle layer, which effectively captures multi-scale features in high-speed flows, while the bottleneck of 20 nodes at both ends helps filter energy dissipation noise at low speeds.

However, it is noteworthy that all models show a significant drop in predictive performance (R^2^ < 0.71) at 50 m/s, suggesting that purely data-driven approaches have inherent limitations in modeling complex physical processes—such as elastic–plastic deformations and material failures—associated with low-velocity ballistic trajectories.

Based on our comprehensive experimental analysis, we propose the following optimal strategy for architecture selection: the ResNetMDN is ideal for the low-velocity range (50–90 m/s) due to its robustness to measurement noise and uncertainty. It should also be used in the medium velocity range (95–144 m/s) to achieve the best prediction accuracy (R^2^ > 0.9). For velocities exceeding 144 m/s, particularly in ultra-high-speed conditions near 200 m/s, the five-layer asymmetric structure is recommended for the most reliable predictions.

**Figure 7:**
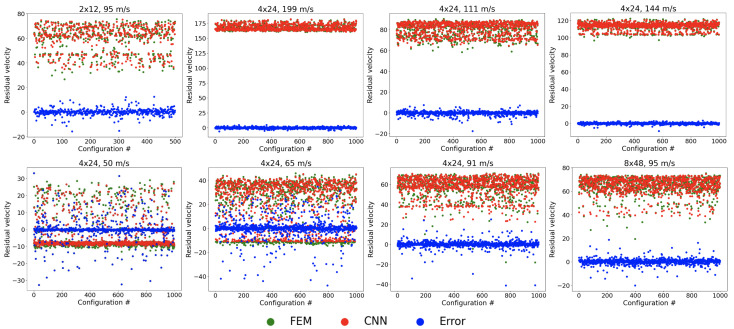
Overview of errors in projectile residual velocity prediction.

Figure 7 presents the performance of the CNN in predicting the projectile residual velocity. The error distribution demonstrates strong convergence, with most prediction errors clustered near zero, indicating high accuracy under typical conditions. However, the model exhibits notable performance degradation at the lower initial velocities of 50 m/s and 65 m/s.

This discrepancy arises from a distinct physical behavior at low velocities: rather than fully penetrating the target, projectiles frequently undergo elastic rebound, leading to negative residual velocities. As illustrated in Figure 6, this phenomenon produces a bimodal data distribution—one peak corresponding to successful penetration (positive velocities) and another to rebound (negative velocities). Such imbalance complicates model training, as the CNN struggles to establish a consistent relationship between input features and output velocities. Consequently, the prediction accuracy declines significantly in these low-velocity regimes.

**Figure 8:**
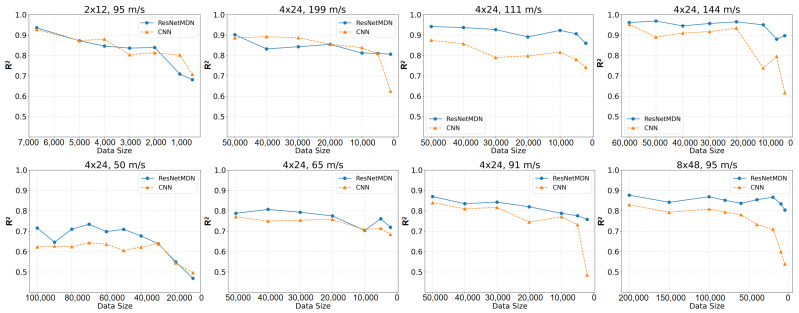
Efficiency (R^2^ score) of CNN and ResNetMDN depending on the dataset size for the impact problem. To highlight the decline in model fitting performance with decreasing dataset size, the figure is arranged in a right-to-left order.

In Figure 8, we show the evaluation of the performance of the two models, CNN and ResNetMDN, using training sets of different sizes, with the coefficient of determination (R^2^) as the evaluation metric. R^2^ measures the model’s predictive accuracy, with values closer to 1 indicating better performance. By comparing model performance across various dataset sizes, we can assess how the data volume influences the predictive capabilities of the models.

The experimental setup included variations in the target sizes (e.g., 2 × 12, 4 × 24, 8 × 48) and impact velocities (e.g., 199 m/s, 144 m/s, 111 m/s, 95 m/s, 91 m/s, 65 m/s, and 50 m/s). These factors introduce different levels of complexity into the simulated data, affecting the models’ ability to fit and predict accurately. In each plot, the horizontal axis represents the dataset size, while the vertical axis shows R^2^, with higher values reflecting better model performance. The results show that the ResNetMDN (blue line) consistently outperforms the CNN (orange line) across all dataset sizes, particularly when the dataset is small. This suggests that the ResNetMDN has superior learning capabilities, enabling it to extract useful features even from limited data and improve its predictions. In contrast, the CNN’s performance is more modest with smaller datasets, indicating weaker modeling ability in such cases.

As the dataset size increases, both models show a trend toward stabilizing performance. When the dataset reaches larger sizes (e.g., 50,000 to 200,000) the performance difference between the two models becomes less pronounced. However, ResNetMDN still maintains a slight edge in certain conditions, suggesting its greater adaptability to complex patterns and its ability to handle the increased diversity present in larger datasets.

### 4.3. Processing Problematic Composite Configurations Using the ANN

Finite element models often face significant challenges when simulating large deformations and high strain rates. These conditions can cause the computational mesh to distort severely, leading to errors or even causing the simulation to stop unexpectedly. Such difficulties are especially common in high-impact scenarios. To address these problems, researchers typically employ techniques such as reducing the simulation time step, adjusting the mesh settings, modifying the contact parameters, altering the material properties, remeshing, or removing highly distorted elements. In some cases, alternative numerical methods, like the material point method (MPM), smoothed particle hydrodynamics (SPH), or peridynamics, are used to overcome the limitations of conventional finite element approaches.

**Figure 9:**
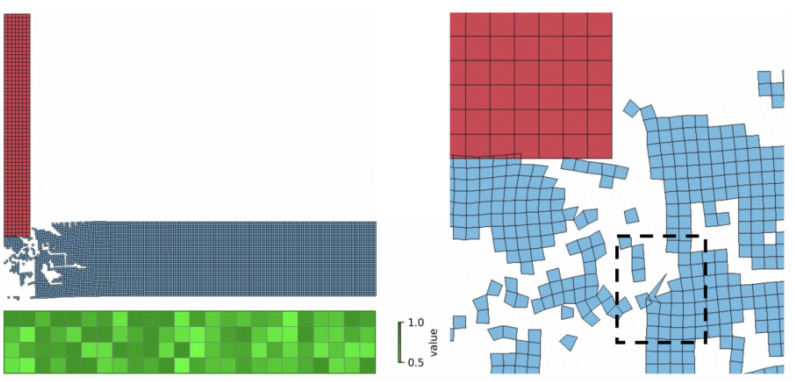
An example of a problematic 4 × 24 cell composite configuration resulting in an excessive element distortion for the 199 m/s projectile velocity. The extreme deformation of the mesh, as highlighted by the dashed rectangle, leads to excessive element distortion. This distortion causes numerical instability and loss of accuracy, ultimately resulting in computational failure of the finite element method due to the inability of the solver to handle severely distorted elements.

In our analysis of impact simulation datasets, certain composite configurations consistently caused the simulation solver to fail due to excessive mesh deformation. For example, in simulations using a 4 × 24 mesh at a projectile velocity of 91 m/s, 40 out of 63,400 cases failed to produce a valid result. This issue became more pronounced at higher velocities; at 199 m/s, 134 out of 56,646 simulations terminated prematurely because of computational errors. Figure 9 shows an example of a problematic 4 × 24 composite configuration and a 199 m/s case. This exact case could be resolved by a reduction in the time step providing 173.3 m/s residual velocity, whereas the ANN predicts 171.6 m/s.

### 4.4. Residual Velocity Prediction for FEM Failures

**Figure 10:**
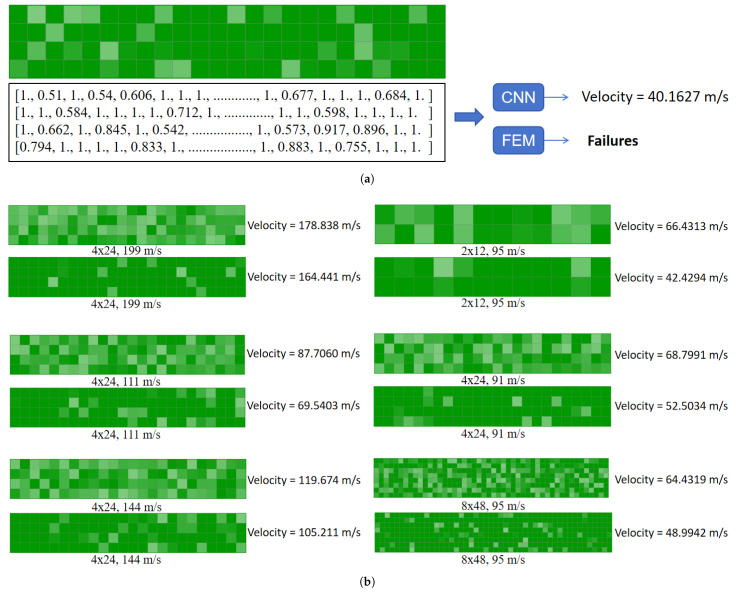
Predicting the FEM application failures based on CNN models. (**a**) illustrates the CNN-predicted residual velocity of the projectile in this perforated plate pattern (4 × 24, 65 m/s). (**b**) depicts the projectile residual velocity prediction results for different patterns of perforated plate conditions.

This section explores the prediction results of the CNN model for the residual velocity of the projectile for a specific perforation pattern. Figure 10a shows the results of the CNN in predicting the residual velocity of a projectile with a 4 × 24 arrangement of perforated plates under the impact of a projectile with an initial velocity of 65 m/s, as well as the distribution of the structural states of each cell. It is worth noting that the FEM calculations failed to converge (labeled as “Failures”) for this case, indicating the limitations of conventional numerical methods in simulating this type of highly nonlinear impact problem. The state data of each cell of the plate are detailed in the figure (represented by normalized values from 0 to 1), where values closer to 1 indicate that the structural integrity of the cell is well maintained, while lower values (e.g., 0.51, 0.542, etc.) may correspond to localized plastic deformations, pore expansions, or areas of material failures.

Figure 10b systematically compares the prediction performance of the CNN model for different perforated plate composite configurations (e.g., 4 × 24, 2 × 12, 8 × 48, etc.) and a wide range of projectile initial velocities (50–199 m/s). By analyzing the difference between the target velocity (e.g., 199 m/s) and the CNN-predicted velocity (e.g., 164.441 m/s), the generalization ability of the model can be assessed under different impact conditions. It is particularly noteworthy that these cases are all complex conditions, where the FEM has difficulty converging, further highlighting the alternative modeling advantages of the CNN in impact problems dominated by high strain rates, large deformations, and the nonlinear behavior of materials.

Table 4 presents the performance comparison of two neural network models, ResNetMDN and CNN, in predicting FEM failure cases under various composite configurations of convolutional channels and impact velocities. Across all composite configurations, the ResNetMDN model consistently outperforms the standard CNN model. For instance, at the 4 × 24 (111 m/s) composite configuration, the ResNetMDN achieves the highest performance score of 0.929 compared to the CNN’s 0.878. Even at the lowest velocity setting (4 × 24, 91 m/s), the ResNetMDN maintains a superior score of 0.825 over CNN’s 0.766. The trend persists at 8 × 48 (95 m/s), where the ResNetMDN scores 0.905, while the CNN achieves 0.823.

These results demonstrate the superior predictive capability of the ResNetMDN architecture, particularly in handling a variety of velocity and convolutional channel settings relevant to FEM failure prediction.

## 5. Conclusions

This research demonstrates that artificial neural networks offer an effective solution for reducing the computational costs in plate impact analyses. By training convolutional neural networks (CNNs) on datasets generated from finite element method (FEM) simulations, we can rapidly predict the impact outcomes with high accuracy. Although generating these datasets initially requires significant computational resources—for example, over 100,000 simulations for the 8 × 48 impact scenario—subsequent predictions using the trained network are nearly instantaneous and no longer depend on expensive or license-restricted FEM LS-DYNA R14.1.0 software. Moreover, the dataset generation process can be fully automated, further lowering the development costs. The CNN-based approach is especially advantageous when a large number of design variations must be evaluated, such as during the prototyping stage, where general trends in structural strength are of primary interest. However, it is important to acknowledge that real-world engineering problems often involve complex combinations of geometric, material, and loading parameters. Each unique scenario may require dedicated analysis to ensure reliable predictions.

Beyond reducing the computation time and cost, the proposed neural network approach addresses several challenges inherent to numerical methods like FEM, including instability from contact interactions and mesh distortion under high strain rates. In our study, certain composite configurations caused the FEM simulations to fail; nevertheless, the neural network was able to provide accurate predictions for these problematic cases by leveraging information from the rest of the dataset. This approach can be extended to a more realistic situation when not only is the target geometry varied (the cell layout as in this case), but also the material data: the initial and boundary conditions.

While the proposed framework demonstrates strong predictive performance for PMMA-based composites under normal (perpendicular) impact conditions, its generalizability to other material systems and impact angles remains untested. This is primarily due to the current training dataset, which only included PMMA composites and perpendicular impact scenarios. As such, the present model’s predictions may not reliably extend to different polymer matrices, fiber reinforcements, or non-normal impact angles without further retraining or transfer learning. Addressing this limitation will require the collection of additional FEM and experimental data for a broader range of material types and loading conditions. Future work will focus on expanding the dataset, exploring transfer learning strategies, and validating the model’s applicability to more complex and diverse impact scenarios, thereby enhancing its practical relevance and generalizability.

In addition to these challenges in expanding the scope of the dataset, another important limitation is the computational cost associated with generating large high-fidelity datasets, especially for complex 3D FEM models. As the diversity and complexity of the data increase, so does the demand on computational resources, which can become a bottleneck for further development. While our current work demonstrates the effectiveness of the proposed hybrid approach with simplified models, scaling to more intricate geometries will require significant computational resources. To overcome this, future work will explore strategies such as transfer learning, active learning, and parallel computation to address these challenges and enable broader applicability.

## Figures and Tables

**Table 1:** Standard and modified PMMA properties.

Material Property	Standard PMMA	Modified PMMA	Steel
Young’s modulus (Pa)	$3.3\times 10^{9}$	[$1.65\times 10^{9}$–$3.3\times 10^{9}$]	$2.08\times 10^{11}$
Poisson’s ratio	0.35	0.35	0.28
Density (kg/$m^{3}$)	1190	1190	7720
Ultimate stress (Pa)	$7\times 10^{7}$	[$35\times 10^{6}$–$70\times 10^{6}$]	-
Incubation time (s)	$2.1\times 10^{-6}$	[$1.05\times 10^{-6}$–$2.1\times 10^{-6}$]	-

**Table 2:** Comparison of the proposed ResNetMDN-FEM framework with traditional regression-based FEM-ANN hybrids.

Aspect	Traditional Regression Models (e.g., MLP, Standard ANN)	Proposed ResNetMDN-FEM Hybrid
Feature Extraction	Shallow architectures; limited capacity for complex feature learning	Deep residual structure enables robust and hierarchical feature extraction
Output Modeling	Predicts single-point estimates (unimodal); limited handling of output variability	Models multimodal outputs via a hybrid density (mixture density network) layer
Uncertainty Quantification	Typically absent or limited; does not explicitly quantify prediction uncertainty	Joint optimization integrates uncertainty quantification directly into predictions
Handling of Failed Cases	Often poorly generalizes to failed FEM cases	Explicitly incorporates failed cases and their distributions via multimodal outputs
Generalizability	Limited, especially in sparse or failure-prone data regimes	Improved, due to richer representations and uncertainty-aware predictions

**Table 3:** Performance comparison of trajectory residual velocity prediction based on different CNN architectures. For example, (20, 40, 80, 40, 20) indicates the number of channels in each of the five layers of the neural network, starting from the input layer to the output layer.

CONV Channel	2×12 (95 m/s)	4×24 (199 m/s)	4×24 (144 m/s)	4×24 (111 m/s)	4×24 (91 m/s)	4×24 (65 m/s)	4×24 (50 m/s)	8×48 (95 m/s)
ResNetMDN	0.936	0.906	0.931	0.928	0.870	0.818	0.704	0.875
(20,20,20)	0.911	0.924	0.936	0.812	0.842	0.775	0.652	0.851
(20,40,20)	0.904	0.919	0.872	0.794	0.836	0.791	0.687	0.867
(40,40,40)	0.918	0.927	0.914	0.856	0.847	0.798	0.665	0.843
(30,30,30)	0.917	0.925	0.947	0.823	0.831	0.782	0.698	0.856
(30,60,30)	0.919	0.901	0.943	0.781	0.850	0.777	0.649	0.861
(60,60,60)	0.917	0.923	0.919	0.869	0.839	0.802	0.673	0.849
(20,20,20,20)	0.920	0.927	0.941	0.845	0.845	0.786	0.659	0.867
(20,40,40,20)	0.918	0.931	0.942	0.803	0.833	0.793	0.681	0.872
(40,40,40,40)	0.910	0.930	0.949	0.877	0.849	0.774	0.667	0.845
(30,30,30,30)	0.931	0.919	0.949	0.828	0.838	0.789	0.692	0.864
(30,60,60,30)	0.925	0.912	0.919	0.792	0.843	0.780	0.644	0.858
(60,60,60,60)	0.927	0.907	0.949	0.863	0.834	0.796	0.677	0.842
(20,20,20,20,20)	0.916	0.911	0.914	0.799	0.852	0.781	0.663	0.870
(20,40,80,40,20)	0.923	0.935	0.955	0.843	0.840	0.800	0.689	0.848
(40,40,40,40,40)	0.893	0.840	0.950	0.894	0.846	0.772	0.656	0.853
(80,80,80,80,80)	0.913	0.913	0.947	0.834	0.837	0.788	0.671	0.859

**Table 4:** Performance of neural network models in predicting FEM failure cases.

CONV Channel	4×24 (199 m/s)	4×24 (111 m/s)	4×24 (144 m/s)	4×24 (91 m/s)	8×48 (95 m/s)
ResNetMDN	0.793	0.929	0.925	0.825	0.905
CNN	0.763	0.878	0.900	0.766	0.823

## Data Availability

The original contributions presented in the study are included in the article, further inquiries can be directed to the corresponding author.
